# Multi Directional Repeated Sprint Is a Valid and Reliable Test for Assessment of Junior Handball Players

**DOI:** 10.3389/fphys.2018.00317

**Published:** 2018-04-04

**Authors:** Amin Daneshfar, Daniel E. Gahreman, Majid S. Koozehchian, Sadegh Amani Shalamzari, Mozhgan Hassanzadeh Sablouei, Thomas Rosemann, Beat Knechtle, Pantelis T. Nikolaidis

**Affiliations:** ^1^School of Health Sciences, University of Canterbury, Christchurch, New Zealand; ^2^College of Health and Human Sciences, Charles Darwin University, Darwin, NT, Australia; ^3^Department of Kinesiology, Jacksonville State University, Jacksonville, AL, United States; ^4^Department of Exercise Physiology, Physical Education and Sport Science Faculty, Kharazmi University, Tehran, Iran; ^5^Central Tehran Branch, Department of Exercise Physiology, Physical Education and Sport Science Faculty, Islamic Azad University, Tehran, Iran; ^6^Institute of Primary Care, University of Zurich, Zurich, Switzerland; ^7^Medbase St. Gallen Am Vadianplatz, St. Gallen, Switzerland; ^8^Exercise Physiology Laboratory, Nikaia, Greece

**Keywords:** exercise testing, muscle strength, speed, team sport, test-retest

## Abstract

The aim of the present study was to examine the validity and reliability of a 10 × (6 × 5 m) multi-directional repeated sprint ability test (RSM) in elite young team handball (TH) players. Participants were members of the Iranian national team (*n* = 20, age 16.4 ± 0.7 years, weight 82.5 ± 5.5 kg, height 184.8 ± 4.6 cm, body fat 15.4 ± 4.3%). The validity of RSM was tested against a 10 × (15 + 15 m) repeated sprint ability test (RSA), Yo-Yo Intermittent Recovery test Level 1 (Yo-Yo IR1), squat jump (SJ) and countermovement jump (CMJ). To test the reliability of RSM, the participants repeated the testing sessions of RSM and RSA 1 week later. Both RSA and RSM tests showed good to excellent reliability of the total time (TT), best time (BT), and weakest time (WT). The results of the correlation analysis showed significant inverse correlations between maximum aerobic capacity and TT in RSA (*r* = −0.57, *p* ≤ 0.05) and RSM (*r* = −0.76, *p* ≤ 0.01). There was also a significant inverse correlation between maximum aerobic capacity with fatigue index (FI) in RSA test (*r* = −0.64, *p* ≤ 0.01) and in RSM test (*r* = −0.53, *p* ≤ 0.05). BT, WT, and TT of RSA was largely-to-very largely correlated with BT (*r* = 0.58, *p* ≤ 0.01), WT (*r* = 0.62, *p* ≤ 0.01), and TT (*r* = *0*.65, *p* ≤ 0.01) of RSM. BT in RSM was also correlated with FI in RSM (*r* = 0.88, *p* ≤ 0.01). In conclusion, based on the findings of the current study, the recently developed RSM test is a valid and reliable test and should be utilized for assessment of repeated sprint ability in handball players.

## Introduction

Exercise testing in handball includes a series of anthropometric and physiological measurements associated with performance in this team sport (Schwesig et al., 2017). The anthropometric and physiological characteristics of team handball (TH) players are relative to their position in the field and competitive level (Nikolaidis et al., 2015b). Elite handball players have significantly higher strength, speed, agility, and cardiovascular endurance than their less competitive counterparts (Mohamed et al., 2009; Nikolaidis and Ingebrigtsen, 2013). In addition, handball players possess a high level of aerobic and anaerobic fitness (Buchheit et al., 2009a,b; Souhail et al., 2010) and repeated sprint ability (RSA) (Okuno et al., 2013). RSA is the ability to run short distances at maximum intensity multiple times with incomplete recovery between sprints (Barbero et al., 2006).

Success in handball, similar to many intermittent team sports, is related to strength, power, speed, and ability to perform repeated high intensity sprints in various directions. Analysis of handball matches showed that 12% of the total game time comprised of sprinting and high intensity running (Chelly et al., 2011). The average duration of each run in a handball competition was 14.4 s with 19.5 s recovery between runs (Chelly et al., 2011). However, most sprints in handball are not performed in a direct line and often include one or multiple change of directions (COD) during defensive and offensive actions. These findings suggest that repeated multi direction sprint ability may be a key discriminating factor in handball performance.

RSA is shown to be related to the athletic performance including the 30 m sprint performance test (Gouthon et al., 2015). In addition, a significant correlation has been reported between anaerobic power with the best time (BT, i.e., the fastest trial among those consisting an RSA test) and total time (TT, i.e., the sum of times of all trials in an RSA test) of a RSA test (Gharbi et al., 2015). Anaerobic power of handball players was also correlated with the fatigue index (FI) during a RSA test (Gharbi et al., 2015). Regular RSA training has been shown to improve time in 10 m sprint, height in countermovement jump (CMJ) and speed of ball in jump shooting in handball players (Dello Iacono et al., 2016). Therefore, different variations of RSA could be utilized in training and assessment of athletic performance in TH players.

RSA protocols vary in the number of sprints, distance of each sprint, recovery between sprints, and the use of COD (Mokou et al., 2016). Independent from these variables, the main indices of an RSA test are BT, TT, and FI (Atkinson et al., 2003). Most RSA tests include multiple sprints in a straight line or with only one COD. Therefore, straight RSA tests may not be suitable for the assessment of athletic performance in sports that include multiple changes of directions during a real match.

Recently, a multi-directional RSA test (RSM) has been developed and examined for validity and reliability in basketball players (Padulo et al., 2016). RSM includes multiple sprints with lateral movements as well as straight line movements and has more face validity than the RSA test protocols for basketball and handball, as it has been shown that these team sports include a large number of lateral movements in addition to straight-line sprints (Taylor et al., 2017). Since anthropometric and physiologic characteristics of basketball players are not the same as handball players (Jiménez et al., 2009), the reliability and validity of RSM should be assessed with handball players. The aim of the present study was to examine the validity and reliability of an RSM test in elite young handball players.

## Materials and methods

### Study design and subjects

**Figure 1:**
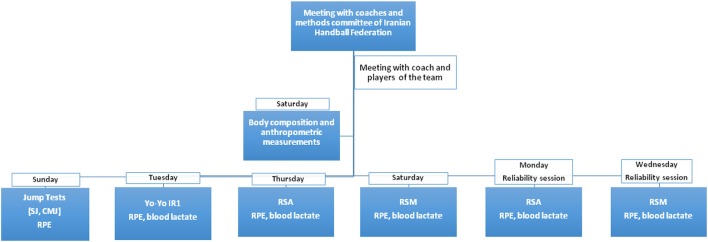
Experimental design of the study. SJ, squat jump; CMJ, countermovement jump; RPE, rate of perceived exertion; Yo-Yo IR1, Yo-Yo intermittent recovery test level 1; RSA, repeated sprint ability test; RSM, multi-directional repeated sprint ability test.

The design of the current study was a single group cross over in which participants completed two RSA and two RSM tests in random orders (Figure 1). Twenty handball players (age: 16.4 ± 0.7 years, sport experience 5.6 ± 2.7 years, weekly training volume 9 h) of the Iranian national team volunteered to participate in this study (Table 1). This study was carried out in accordance with the recommendations of the Iranian Handball Federation and National Olympic Committee of Iran. Participants' parents or guardians provided written informed consent. All procedures were in accordance with the Declaration of Helsinki. The protocol was approved by the Ethics Committee of the Iranian Handball Federation.

**Table 1:** Anthropometric characteristics, jumping performance and aerobic capacity of participants.

**Parameter**	**Mean ± SD**
Age (years)	16.4 ± 0.7
Body mass (kg)	82.5 ± 5.5
Height (cm)	184.8 ± 4.6
Body fat (%)	15.4%
VO_2_max (mL.min^−1^.kg^−1^)	47.5 ± 2.8
RPE (a.u.)	9.3 ± 0.4
SJ (cm)	29.70 ± 4.89
CMJ (cm)	32.56 ± 4.54
RSM-lactate (mmol.L^−1^)	10.50 ± 0.28
RSA-lactate (mmol.L^−1^)	9.99 ± 0.15

*SD, standard deviation; VO_2_max, maximal oxygen uptake, estimated by covered distance in Yo-Yo IR1; RPE, rate of perceived exertion after Yo-Yo IR1; a.u., arbitrary units; SJ, squat jump; CMJ, countermovement jump; RSM, multidirectional repeated sprint ability test; RSA, straight-line repeated sprint ability test*.

### Measuring protocol

To avoid the diurnal variation of performance, assessments were completed between 4:30 p.m. and 6:00 p.m. in ambient temperature of 25°C and relative humidity of 40% at the national training hall. To minimize the effect of familiarization on athletic performance, each participant completed two RSA and RSM trials 48 h prior to data collection. Participants' height and weight were measured using a standard stadiometer and a calibrated scale. Body fat (%) was estimated using bioelectrical impedance (InBody 220, Phymed, Korea).

The vertical displacement of participants during squat jump (SJ) and CMJ was evaluated using accelerometer MyotestTM (Myotest SA, Sion, Switzerland) which was valid and reliable equipment to assess jumping performance (Choukou et al., 2014). Two trials were performed for each test and the best score was recorded. One-minute break was allowed between trials and between SJ and CMJ. Both tests were performed with participants maintaining hands on their hips according to the protocol of Bosco and Rusko (1983).

Yo-Yo intermittent test level 1 and Bangsbo formula was used to assess the aerobic capacity of participants (Bangsbo et al., 2008). This test has been recommended as more sensitive measure of changes in performance than maximum oxygen uptake in team sports (Bangsbo et al., 2008) and has been used in handball (Hermassi et al., 2017b), soccer (Eniseler et al., 2017) and basketball (Padulo et al., 2016). Participants were running between two lines separated by 20 m continuously at an incremental pace, dictated by an audio signal, till exhaustion. This test differs from 20 m shuttle run test (Batista et al., 2017) as it includes a 10 s recovery after the completion of each 40 m.

**Figure 2:**
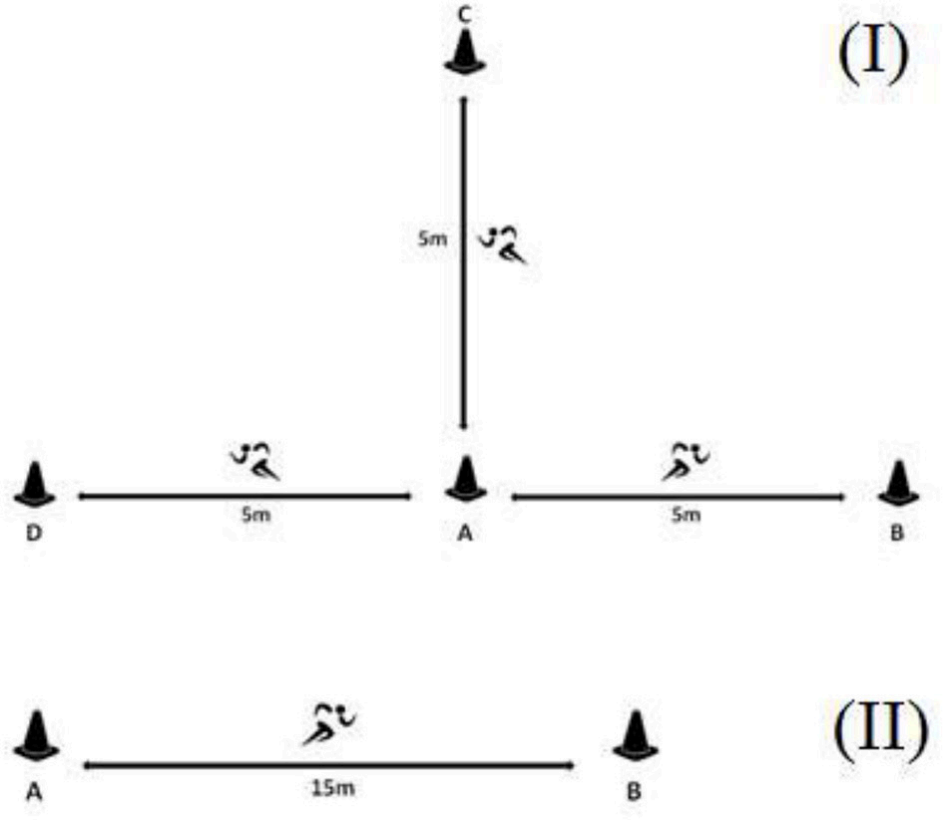
Presentation of the RSM **(I)** and RSA test **(II)**. RSA, straight-line repeated sprint ability test; RSM, multi-directional repeated sprint ability test.

Each testing session started with a 15 min warm-up including running at low-to-moderate intensity and dynamic stretching. The RSA test included ten 30 m sprints with one COD (i.e., 15 + 15 m) and 30 s passive recovery between trials (Padulo et al., 2016). The RSM test consisted of ten 30 m sprints with multiple CODs (Figure 2) and 30 s passive recovery between trials (Padulo et al., 2016). Weakest time (WT) was defined as the slowest time among trials. Each trial was recorded in the nearest 0.01 s using a pair of photocells (Newtest Oy, Oulu, Finland). FI of RSA and RSM (Atkinson et al., 2003) was calculated using the Fitzsimons' formula (Fitzsimons et al., 1993).

A drop of blood was collected from the index finger before and 3 min after each testing session to analyse the blood lactate concentration using a portable lactate monitor (Accusport Lactate Meter, Boehringer Mannheim®, and Germany). At the end of each testing session, participants rated the intensity of the testing session using the modified 10-points Borg scale (Foster et al., 2001). To allow sufficient recovery and prevent fatigue accumulation between testing sessions, there was a 48 h rest between each testing session.

#### Statistical analysis

Data were analyzed using SPSS 24.0 (SPSS Inc., Chicago, IL). The probability level of statistical significance was set at *p* ≤ 0.05 and descriptive statistics were expressed as means ± SE. Intra-class Correlation Coefficient (ICC) estimates and their 95% confident intervals were calculated based on a single-rating (*k* = 1), absolute-agreement, 2-way mixed-effects model (McGraw and Wong, 1996). Based on the 95%confident interval of the ICC estimate, values less than 0.5, between 0.5 and 0.75, between 0.75 and 0.9, and greater than 0.90 are indicative of poor, moderate, good, and excellent reliability, respectively (Koo and Li, 2016). A will Hopkins Typical Error of Measurement, with Pearson's correlation were implemented to determine the agreement between measurements (Hopkins, 2016). To assess the size and direction of the linear relationship between BT, WT, TT, BLa, and FI, a bivariate Pearson's product-moment correlation coefficient (*r*) was calculated.

## Results

The anthropometric characteristics, jumping performance and aerobic capacity of participants can be seen in Table 1. The results of reliability measurements using ICC are presented in Table 2. According to the results, both RSA and RSM tests showed good to excellent reliability of the TT, BT and WT. In addition, the results of the agreement between the measurements of both RSA and RSM were analyzed using Will Hopkins Typical Error of Measurement and are presented in Table 3.

**Table 2:** Reliability of measurements in RSA and RSM using ICC.

		**RSA**				**RSM**			
		**Mean ± SE**	**ICC**	**95% CI**		**Mean ± SE**	**ICC**	**95% CI**	
TT (s)	Test	68.97 ± 0.23	0.83	0.57	0.93	112.17 ± 0.61	0.81	0.54	0.93
	Re-test	69.25 ± 0.24				112.59 ± 0.68			
BT (s)	Test	6.35 ± 0.08	0.88	0.70	0.95	9.99 ± 0.09	0.99	0.97	1.00
	Re-test	6.30 ± 0.08				1.02 ± 0.08			
WT (s)	Test	7.24 ± 0.07	0.82	0.54	0.93	11.86 ± 0.19	0.91	0.77	0.96
	Re-test	7.23 ± 0.08				11.97 ± 0.17			
FI (%)	Test	9.07 ± 1.15	0.99	0.97	1.00	12.34 ± 0.84	0.78	0.47	0.91
	Re-test	9.32 ± 1.06				11.44 ± 0.54			
Lactate (mmol.L^−1^)	Test	9.99 ± 0.15	0.86	0.63	0.95	10.50 ± 0.28	−0.06	−1.34	0.56
	Re-test	9.77 ± 0.16				11.12 ± 0.22			
RPE (a.u.)	Test	8.80 ± 0.13	0.94	0.85	0.98	8.60 ± 0.12	0.93	0.82	0.97
	Re-test	8.76 ± 0.10				8.58 ± 0.12			

*RSA, straight-line repeated sprint ability test; RSM, multidirectional repeated sprint ability test; ICC, intra-class correlation coefficient; TT, total time; BT, best time; WT, weakest time; FI, fatigue index; RPE, rate of perceived exertion*.

**Table 3:** Typical Error of Measurement and correlation of measurements in RSA and RSM using Will Hopkins reliability test.

		**RSA**				**RSM**			
		**Estimate**	**Lower CL**	**Upper CL**	***r***	**Estimate**	**Lower CL**	**Upper CL**	***r***
TT (s)	Raw units	0.74	0.56	1.09	0.72	2.04	1.54	3.01	0.69
	Standardized	0.96	0.53	2.23		1.06	0.58	2.67	
BT (s)	Raw units	0.23	0.17	0.34	0.79	0.08	0.06	0.011	0.98
	Standardized	0.78	0.45	1.61		0.19	0.12	0.32	
WT (s)	Raw units	0.23	0.17	0.33	0.69	0.48	0.36	0.70	0.83
	Standardized	1.05	0.57	2.62		0.66	0.39	1.26	
FI (%)	Raw units	1.09	0.83	1.62	0.98	2.66	2.01	3.93	0.72
	Standardized	0.21	0.13	0.35		0.96	0.53	2.23	
Lactate (mmol.L^−1^)	Raw units	0.42	0.32	0.62	0.79	1.27	0.96	1.88	−0.03
	Standardized	0.78	0.45	1.60		29.96	2.19	1.88	
RPE (a.u.)	Raw units	0.25	0.19	0.37	0.91	0.29	0.22	0.43	0.86
	Standardized	0.44	0.27	0.77		0.60	0.35	1.10	

*RSA, straight-line repeated sprint ability test; RSM, multidirectional repeated sprint ability test; TT, total time; BT, best time; WT; weakest time; FI, fatigue index; RPE, rate of perceived exertion*.

**Figure 3:**
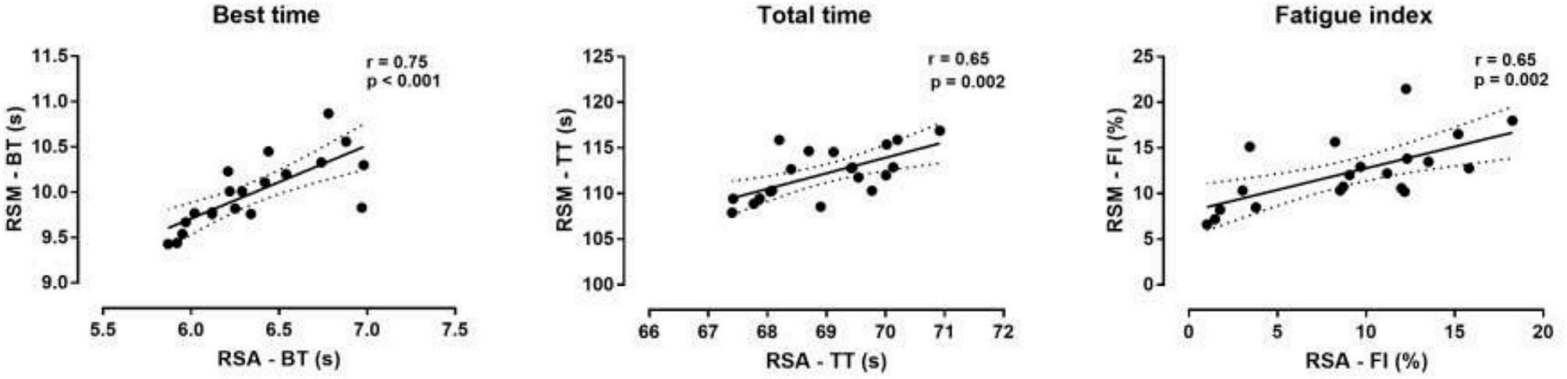
Best time, total time and fatigue index in RSM and RSA. RSA, repeated sprint ability test; RSM, multi-directional repeated sprint ability test.

**Figure 4:**
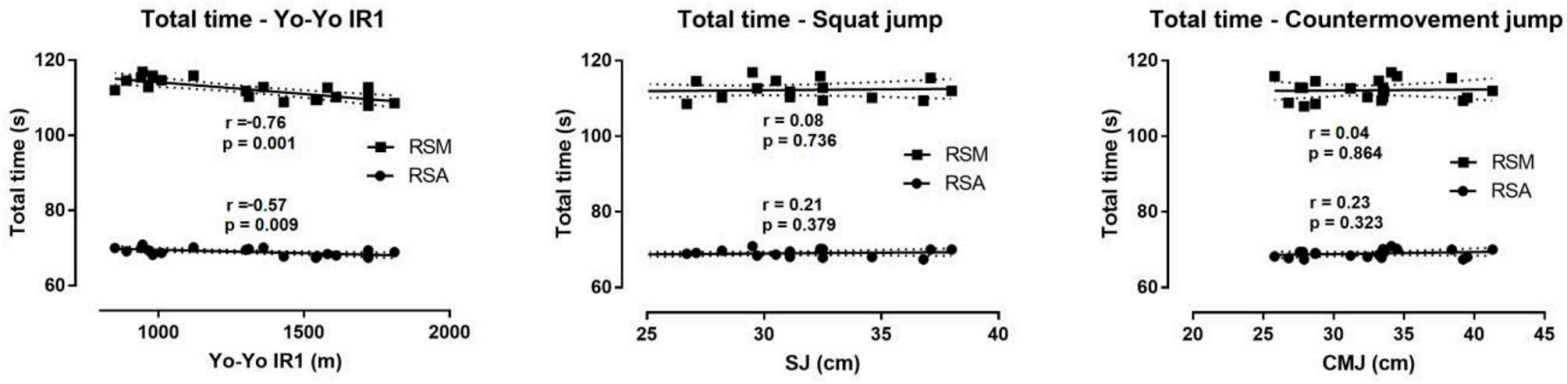
Relationship of total time in RSM and RSA with jumping performance and aerobic capacity. SJ, squat jump; CMJ, countermovement jump; RPE, rate of perceived exertion; Yo-Yo IR1, Yo-Yo intermittent recovery test level 1; RSA, repeated sprint ability test; RSM, multi-directional repeated sprint ability test.

The results of the correlation analysis showed significant inverse correlations between maximum aerobic capacity and TT in RSA (*r* = −0.57, *p* ≤ 0.05) and RSM (*r* = −0.76, *p* ≤ 0.01). There was also a significant inverse correlation between maximum aerobic capacity with FI in RSA test (*r* = −0.63, *p* ≤ 0.01) and in RSM test (*r* = −0.62, *p* ≤ 0.01). In addition, the levels of lactate after RSA and RSM were correlated with maximum aerobic capacity (*r* = 0.49, *p* ≤ 0.05) and (*r* = 0.47, *p* ≤ 0.05) respectively. BT, WT, and TT of RSA were largely-to-very largely correlated with BT, WT, and TT of RSM (Figure 3, Table 4). TT in RSM correlated very largely with the Yo-Yo, but not with SJ and CMJ (Figure 4).

**Table 4:** Correlations between RSM, RSA, jumping performance and aerobic capacity.

	**RSM-BT**	**RSM-FI**	**RSA-TT**	**RSA-BT**	**RSA-FI**	**SJ**	**CMJ**	**Yo-Yo IR1**
RSM-TT	0.660^**^	0.678^**^	0.648^**^	0.576^**^	0.754^**^	0.080	0.041	−0.756^**^
RSM-BT	–	0.880^**^	0.532^*^	0.750^**^	0.698^**^	−0.052	0.030	−0.615^**^
RSM-FI		–	0.518^*^	0.591^**^	0.653^**^	0.112	0.193	−0.529^*^
RSA-TT			–	0.664^**^	0.599^**^	0.208	0.233	−0.571^**^
RSA-BT				–	0.646^**^	0.077	0.108	−0.607^**^
RSA-FI					–	0.136	0.178	−0.641^**^
SJ						–	0.954^**^	−0.165
CMJ							–	−0.170

* *p < 0.05*,

** *p < 0.01; RSA, straight-line repeated sprint ability test; RSM, multidirectional repeated sprint ability test; TT, total time; BT, best time; FI, fatigue index; SJ, squat jump; CMJ, countermovement jump; Yo-Yo IR1, Yo-Yo intermittent recovery test level 1*.

## Discussion

The main findings of the present study were that RSM indices showed a good reliability for both TT and FI and an excellent reliability for BT, WT, and RPE. These results indicate that RSM is a reliable test for the assessment of sprint ability with multiple COD s in youth handball players. TT, BT, WT, and BL showed a good reliability in RSA test. In addition, the reliability of FI and RPE were excellent in RSA test. These results indicate that RSA is a reliable test for assessment of afore-mentioned variables in youth handball players. The RPE levels in both RSA and RSM were similar and near maximal effort suggesting that athletes performed at the maximal level during both tests. The higher reliability of BT and WT in RSM compared to RSA suggest that strongest and weakest sprint performance of athletes are more consistent during RSM that RSA. Therefore, the BT and WT in RSM could be utilized to monitor RSA of elite handball players.

Previous researchers reported a poor reliability for FI when compared to BT and TT (Austin et al., 2013) and have questioned the use and the value of reporting FI in RSA (Oliver, 2009). The findings of the current study supported the claim that reliability of FI, calculated using the Fitzsimons' formula, may not be as strong as TT, BT, and WT in RSM. However, the reliability analysis of FI showed a good reliability in RSM and excellent reliability in RSA. Therefore, it appears that the value of FI in assessment of RSA may be subjective and perhaps specific to variables of a RSA test.

Performance in TT during RSA and RSM were significantly correlated with the maximum aerobic capacity that was assessed by a Yo-Yo test. The higher correlation of RSM with maximum aerobic capacity could be explained by the longer duration of the RSM test. The average TT in RSM was 112 s, twice as much as the duration of the RSA test. Therefore, athletes with higher maximum aerobic capacity could recover better during the sprints and achieve better results. This claim is supported by the significant inverse correlation of maximum aerobic capacity with FI in both RSA and RSM tests. Athletes with a higher maximum aerobic capacity showed quicker recovery between sprints and demonstrated lower FI and blood lactate level.

The results of BT, WT, and the TT of RSM trials were strongly correlated with the BT, WT and the TT of RSA tests. These strong correlations suggest that RSM test is as strong discriminator as RSA for assessing the athletics performance. Considering that RSM is a multi-direction test that stresses athletes' body for a longer period than RSA, it is possible that RSA performs better as a discriminating assessment for athlete selection, talent identification, and evaluation of a training intervention. On the contrary, RSM indices did not correlate with jumping ability, which was in disagreement with previous research showing low-to-moderate relationship between jumping tests and 10 × 20 m RSA test (Nikolaidis et al., 2015a). An explanation of this discrepancy might be the longer duration of RSM in the present study compared to the RSA protocol used in the abovementioned study.

Participants of the current study achieved the maximum aerobic capacity of 47.46 ± 2.76 ml.kg^−1^.min^−1^. This finding suggests that aerobic capacity of youth handball players were at the medium range and slightly above non-athletic populations. A previous study comparing handball players of different performance levels showed no difference for aerobic capacity, whereas the elite players had superior anaerobic power and jumping ability (Nikolaidis and Ingebrigtsen, 2013). Our findings were in agreement with previous studies (Malacko et al., 2013) and suggests that anaerobic energy system is a dominant energy system in TH and success in this sport may be more dependent on anaerobic glycolysis than maximum aerobic capacity.

Since the ability to perform movements in different directions and within short distance is unique in handball, the results of this study should be generalized with caution to other team sport with different characteristics. For instance, the RSM should also be tested by a future study for validity and reliability in soccer due to larger distances covered in-line. Strength of the study was that it confirmed the validity and reliability of the RSM which was developed initially in basketball (Padulo et al., 2016). Considering the popularity of handball, the findings of the present study are of great practical value for coaches and fitness trainers in the context of the training and testing of their players.

The present study established the validity and reliability of RSM test in handball; thus, coaches and fitness trainers are encouraged to use this test to monitor performance of their players. Two training strategies have been recommended to optimize RSA in handball players with the first one relying on the training specificity concept and the second one focusing on the main factors associated with this physical fitness component (Bishop et al., 2011). Accordingly, these strategies could apply in the case of RS; thus, training should include drills similar as RSM, e.g., 10 trials of 5 m distance multi-directional sprints with 30 s passive recovery. In addition, training should also include high-intensity intermittent aerobic exercises using COD similar as the exercises included in the Yo-Yo IR1 test, considering the large-to-very large correlations between all RSM indices and Yo-Yo IR1.

In conclusion, based on the findings of the current study, the novel RSM test is a valid and reliable test and should be utilized for assessment of RSA of handball players. So far, handball professionals use isokinetic strength of knee flexors and extensors, one repetition maximum of half squat, 5 m sprint test, agility and jump tests, Yo-Yo test and in-line RSA to monitor physical fitness (Hermassi et al., 2017a; Maurelli et al., 2017; Schwesig et al., 2017). We recommend the further use of the RSM in handball players in the context of a physical fitness battery administration.

## Author contributions

AD conceived the idea of this paper and PN wrote the first draft. AD, MK, SA and MH participated in the organization of the experimental setting and the data collection. AD, DG, TR, and BK wrote the final draft. All coauthors approved the final version.

### Conflict of interest statement

The authors declare that the research was conducted in the absence of any commercial or financial relationships that could be construed as a potential conflict of interest.
